# Lieutenant-Colonel Isidore Benadotte Lyon

**Published:** 1911-05-06

**Authors:** 


Lieutenant-Colonel Isidore Benadotte Lyon, C.I.E., (I.M.S.), has died in London. He took his M.R.C.S. in 1860, and the L.S.A. in 1863, went out to India, where he held appointments at Bombay, attaining the rank of Brigadier-Surgeon. He was on the retired list at the time of his death.

